# Medical waste management – how industry can help us to protect environment and money?

**DOI:** 10.1080/0886022X.2020.1774900

**Published:** 2020-06-12

**Authors:** Paweł Żebrowski, Jacek Zawierucha, Tomasz Prystacki, Wojciech Marcinkowski, Jolanta Małyszko

**Affiliations:** a Department of Nephrology, Dialysis and Internal Medicine, Medical University of Warsaw, Warsaw, Poland; b Fresenius Medical Care AG und Co KGaA, Fresenius Medical Care, S.A, Polska; c Fresenius Medical Care AG und Co KGaA, Fresenius Nephrocare Polska sp. z o.o.

**Keywords:** Medical waste management, hemodialysis, environment protection, cost

## Abstract

**Background:**

The global climate change and its consequences force us to remodel our processes and rethink the current model of providing the HD treatments. Waste management have a massive impact on the environment and the economy. Every HD session produces above 1 kg of medical waste, which should be properly stored and destroyed. In particular in the pandemia time we should improve the dialysis unit budget as well as decrease CO_2_ emission produced during the waste elimination.

**Materials and Methods:**

The checked the weight of different dialyzers used regularly in dialysis centers in Poland. The Kern CM 320-IN scale was used for the measurement. The measurement accuracy was 0.1 g. Also the filling volume of each dialyzer has been taken into consideration.

**Results:**

The dialyzers were divided into four groups depending on the surface. 1,4m2 in group one, 1.5–1.6 m^2^ in group two, 1.7–1.8 m^2^ in group three and finally 2.0–2.2 m^2^ in group four. FX class dialyzers were lightest in every group. The heaviest ones were Polyflux dialyzers. The difference between the lightest and heaviest dialyzers was about 95 g. The filling volume was lowest in FX dialyzers and the highest in Elisio dialyzers. The difference was 20 mL.

**Conclusions:**

The weight of different dialyzers available on the market differs. The decision-makers should take into account this fact as the additional quality feature. In extreme cases the weight difference reaches 95 g. In yearly perspective, the usage of the lighter dialysis set can cause the 17 million kg decrease of medical waste and significant savings.

Dear Editor,

Medical waste management has become a serious problem. During every hemodialysis session more than 1 kg of medical waste are produced. Part of them are recognized as the infectious waste (waste contaminated with blood and other bodily fluids), according to the World Health Organization [1]. Dialyzers, bloodlines, needles are the most important hazardous waste. They should be properly stored and destroyed. On the basis on projection made by Liyanage et al. [2] in 2010 more than 2.61 million patients were treated with dialysis (both, peritoneal dialysis and hemodialysis) and will double in 2030. The majority of the patients are treated with hemodialysis mode [3]. It shows that about 2.7 million patients get the 156 hemodialyses annually. During 420 millions HD sessions worldwide yearly more than 420 million kg of medical waste are produced. Waste incineration of such big mass has a significant impact on the environment as well as the cost of the treatment. The cost of destroying medical waste is growing almost every day – due to the more restrictive requirements for Waste Disposal Services, costs of energy used for incineration, etc. In the European countries the cost of utilization of 1 kg of medical waste is about 3 Euro [4]. In this study, we assessed the weight of different dialyzers available on the Polish market. The dialyzer weight is the heaviest part of dialysis set influencing significantly the cost of utilization of hazardous medical waste.

The authors checked the weight of different dialyzers regularly used in dialysis centers in Poland. The dialyzers were divided into four groups depending on the surface. 1.4 m^2^ in group one, 1.6–1.7 m^2^ in group two, 1.8–1.9 m^2^ in group three and finally 2.0–2.2 m^2^ in the group four. The Kern CM 320-IN scale was used for the measurement. The measurement accuracy was 0.1 g. The results of the measure were compared with available manufacturer data presented on the official websites. Also the filling volume of each dialyzer has been taken into consideration.

As shown in Table 1 the weight difference between dialyzer produced by different manufacturers is 95 g from the heaviest to lightest ones. The lightest dialyzers are Fresenius FX class filters, the heaviest ones are Baxters Polyflux. The main reason of such a big difference is the wall thickness and the construction of the dialyzer. The material used for dialysis production also plays an important role. The housing of the lightest dialyzer is polypropylene, whereas the housing of most of the other dialyzers is polycarbonate. The density of polycarbonate is approximately 20% greater than the density of polypropylene and that difference in density will be an important contributor to the difference in weight. The weight difference of the dialyzers shows, that its mass should be taken into consideration, when we are choosing the type of the filter. Of course, the most important are still the parameters of the dialyzer (clearances, sieving coefficients), but in case of comparable performance data, the weight can be tip the balance during decision making. The dialysis center treated 100 patients can save 1.482 kg of medical waste yearly only by using the lightest dialyzers, in comparison with the heaviest ones. In a global perspective the savings are much more noticeable. The authors also noticed the difference in priming volume of checked dialyzer. The biggest one was in Elysio dialyzers, the smallest in FX series and Leoceed ones. It can influence on the total weight of medical waste, especially in case of careless emptying the dialysis sets. Besides the blood volume, the dialysis fluid compartment volume of the dialyzer may impact on the waste weight. Manufacturers try to decrease the priming volume to achieve the most effective usage of dialysis fluid. This parameter and its correlation with blood volume are unpublished in official materials issued by dialysis disposables manufacturers and suppliers. Cost (both, environmental and financial) of proper medical waste incineration is an increasingly serious burden for healthcare providers and governments of individual countries. Renal replacement therapy, as the one of the biggest source of hazardous waste, also can be also the pioneer of the new trends in pro-environmental thinking. The dialyzer weight is the heaviest part of dialysis set and its mass significantly influences on the cost of utilization of hazardous medical waste. There are three crucial points to decrease the quantity and mass of the waste – segregation, emptying the fluid remained in dialysis sets and paying attention to the mass of disposables used.

**Table 1.** The results of the weight measure of different dialyzers.

Group	Dialyzer	Surface [m^2^]	Producer	Membrane	Priming volume [mL]	Weight (measured) [g]	Weight (published on the website) [g]
Group I (1.4 m^2^)	Polyfux 14 L	1.4	Baxter	Polyamix	94	168	No data
	Polyflux 140 H	1.4	Baxter	Polyamix	94	203	No data
	FX8	1.4	FMC	Helixone	74	105	No data
	FX60 classix	1.4	FMC	Helixone	74	107	107
	FX60 cordiax	1.4	FMC	Helixone	74	107	105
Group II (1.6–1.7 m^2^)	Leoceed 16N	1.6	Asahi	Polysulfone	86	130	130
	Leoceed 16H	1.6	Asahi	Polysulfone	86	130	130
	Polyflux 17L	1.7	Baxter	Polyamix	115	190	No data
	Polyflux 170H	1.7	Baxter	Polyamix	115	215	No data
Group III (1.8–1.9 m^2^)	Elysio 19L	1.9	Nipro	Polynephron	115	150	No data
	Elysio 19H	1.9	Nipro	Polynephron	115	154	No data
	Leoceed 18N	1.8	Asahi	Polysulfone	96	140	140
	Leoceed 18H	1.8	Asahi	Polysulfone	96	140	140
	FX10	1.8	FMC	Helixone	95	123	123
	FX80 classix	1.8	FMC	Helixone	95	123	123
	FX80 cordiax	1.8	FMC	Helixone	95	123	123
Group IV (2.0–2.2 m^2^)	Polyflux 21L	2.1	Baxter	Polyamix	125	230	No data
	Polyflux 210H	2.1	Baxter	Polyamix	125	230	No data
	Elysio 21L	2.1	Nipro	Polynephron	128	164	No data
	Leoceed 21N	2.1	Asahi	Polysulfone	108	150	150
	Leoceed 21H	2.1	Asahi	Polysulfone	108	150	150
	FX100 classix	2.2	FMC	Helixone	115	135	135
	FX100 cordiax	2.2	FMC	Helixone	115	135	135

Successful implementation of these three points needs the change of behavior and way of thinking of medical staff as well as development of more environment friendly (lighter, recyclable, free of toxic components, like phtalanes) disposables by medical industry. Every healthcare services provider should have in place SOP (standard operating procedure) which describes how to proceed with the waste produced during medical intervention. Firstly, all waste, used in dialysis should be recognized and differentiated as hazardous and nonhazardous ones. Next, nonhazardous waste should be divided into recyclable (paper and plastic) and nonrecyclable waste – but still nonmedical (municipal waste). Only hazardous (dialyzer, bloodline, needles, syringes, etc.) waste will be considered as medical waste [4].

Emptying the dialysis sets from residual fluids also lead to decrease the weight of medical waste and may lead to significant savings [5]. The manufacturers effort should lead to development HD machines which automatically empty the sets. Fresenius 6008 machine allows to save 150 g of medical waste due to the emptying process at the end of dialysis in comparison with Fresenius 4008 and 5008 machines [6]. Also medical staff should take care on fully emptying of the set after dialysis session. This is in line with the ERA-EDTA policy of going green [7].

The last but not least topic is taking into consideration the disposables weight. As it was shown, the mass difference between dialyzers can reach 95 g. Dialyzers are only relatively small (10–20% of total weight of medical waste) part of medical waste. Every part of dialysis set (bloodlines, syringes, sharps, dressings, etc.), treated as hazardous waste is equally important to limit the mass of medical waste. Decreasing the weight of disposables should be the challenge for medical industry – the manufacturer’s efforts in this matter will be appreciated.

Realization of all recommendations listed above can bring remarkable money savings and invaluable benefits for our environment. The strict respect to the waste management policy makes more than 5 kg of medical waste difference in comparison with “careless max” policy – without any differentiation and emptying [4]. In each country both low and high income the cost should be taken into account as well as reimbursement for dialysis should promote environment friendly disposables. Careful proceeding with the disposables (proper procedures, medical staff training and awareness) will directly help to make the HD treatment more cost-effective and help to protect our planet. In addition, in the era of coronavirus disease of 2019 (COVID-19) the spread of the infection should be considered and amount of medical waste is of utmost importance as it generates the extra costs.

## References

[1] World Health Organization 2018. Health-Care waste. Available from: https://www.who.int/news-room/fact-sheets/detail/health-care-waste. Date of entry: May 10th, 2020.

[2] Liyanage T, Toshiharu N, Jha V, et al. Worldwide access to treatment for end-stage kidney disease: a systematic review. Lancet. 2015;385(9981):1917–2014. 25777665 10.1016/S0140-6736(14)61601-9

[3] Jain KA, Blake P, Cordy P, et al. Global trends in rates of peritoneal dialysis. J Am Soc Nephrol. 2012;23(3):533–544. 22302194 10.1681/ASN.2011060607 PMC3294313

[4] Piccoli GB, Nazha M, Ferraresi M, et al. “Eco-dialysis: the financial and ecological costs of dialysis waste products: is a 'cradle-to-cradle' model feasible for planet-friendly haemodialysis waste management?” Nephrol Dial Transplant. 2015;30(6):1018–1027. 25808949 10.1093/ndt/gfv031

[5] Connor A, Mortimer F. The green nephrology survey of sustainability in renal units in England, Scotland and Wales. J Ren Care. 2010;36(3):153–160. 20690969 10.1111/j.1755-6686.2010.00183.x

[6] Zawierucha J, Prystacki T, Domański L, et al. Does modern HD machines can improve the waste management? Two centers observational study. Kidney Int Rep. 2020;5:307–308.32154452

[7] Blankestijn PJ, Arici M, Bruchfeld A, et al. ERA-EDTA invests in transformation to greener health care. Nephrol Dial Transplant. 2018;33(6):901–903. 29800317 10.1093/ndt/gfy092

